# Microglia-derived IL-1*β* triggers p53-mediated cell cycle arrest and apoptosis in neural precursor cells

**DOI:** 10.1038/cddis.2015.151

**Published:** 2015-06-04

**Authors:** J Guadagno, P Swan, R Shaikh, S P Cregan

**Affiliations:** 1 Department of Physiology and Pharmacology, University of Western Ontario, London, ON, Canada; 2 Robarts Research Institute, University of Western Ontario, London, ON, Canada

## Abstract

Neurogenesis persists in the adult brain and can contribute to learning and memory processes and potentially to regeneration and repair of the affected nervous system. Deregulated neurogenesis has been observed in neuropathological conditions including neurodegenerative diseases, trauma and stroke. However, the survival of neural precursor cells (NPCs) and newly born neurons is adversely affected by the inflammatory environment that arises as a result of microglial activation associated with injury or disease processes. In the present study, we have investigated the mechanisms by which microglia affect NPC proliferation and survival. Importantly, we demonstrate that interleukin-1*β* (IL-1*β*) produced by lipopolysaccharide/interferon-*γ*-activated microglia is necessary to induce cell cycle arrest and apoptosis in NPCs *in vitro*. Mechanistically, we show that IL-1*β* activates the tumor suppressor p53 through an oxidative stress-dependent mechanism resulting in p53-mediated induction of the cyclin-dependent kinase inhibitor p21 and the proapoptotic Bcl-2 (B-cell lymphoma-2) family members Puma (p53-upregulated modulator of apoptosis) and Noxa. Furthermore, we demonstrate that cell cycle arrest and apoptosis induced by recombinant IL-1*β* or activated microglia is attenuated in *p53*-deficient NPCs. Finally, we have determined that IL-1*β* induces NPC death via the p53-dependent induction of Puma leading to the activation of a Bax (Bcl-2-associated X protein)-mediated mitochondrial apoptotic pathway. In summary, we have elucidated a novel role for p53 in the regulation of NPC proliferation and survival during neuroinflammatory conditions that could be targeted to promote neurogenesis and repair in a number of neurological conditions.

In the adult brain, neural precursor cells (NPCs) generate new neurons that can be integrated into the CNS circuitry to replace damaged or lost neurons, and contribute to learning and memory processes.^1, 2^ Dysregulation of adult neurogenesis has been observed in animal models of stroke and epilepsy, and neurodegenerative diseases including Alzheimer's, Huntington's and Parkinson's disease.^3, 4, 5, 6, 7, 8^ However, the extent to which neurogenesis contributes to brain repair is severely limited by the neuroinflammatory processes associated with these neurological conditions.^9, 10, 11, 12, 13^ Microglia are the resident immune cells of the central nervous system and are the primary regulators of neuroinflammatory responses. During injury and pathological conditions, microglia cells become activated and depending on the nature and duration of the stimulus can produce either anti-inflammatory or proinflammatory factors that can differentially affect neurogenesis.^13, 14, 15, 16^ Microglia cells induced to exhibit a proinflammatory phenotype release cytokines such as TNF*α*, interleukin-6 (IL-6) and IL-1*β* and decrease neurogenesis and NPC survival *in vitro* and *in vivo*.^11, 17, 18^

IL-1*β* is synthesized in microglia as an inactive precursor protein that requires cleavage by caspase-1 (also known as IL-1-converting enzyme or ICE) to be transformed into its mature, biologically active form. IL-1*β* exerts its effects on target cells by binding the cell surface IL-1 type-1 receptor (IL-1R1) leading to the activation of a signaling cascade that results in the activation of mitogen-activated protein kinases and transcriptional regulators such as NF-*κ*B.^19^ Embryonic and adult NPC express IL-1R1 and undergo cell cycle arrest when exposed to IL-1*β in vitro*.^20, 21^ Furthermore, it has been shown that hippocampal neurogenesis is impaired in mice chronically exposed to IL-1*β in vivo* and that IL-1*β*-induced inhibition of hippocampal progenitor cell proliferation was blocked by the IL-1R1 antagonist IL-1RA.^22, 23^ Similarly, it has been demonstrated that hippocampal neurogenesis is markedly reduced in transgenic mice engineered to inducibly express human IL-1*β* in the hippocampus.^24^ Although these data demonstrate that IL-1*β* has antineurogenic properties, the mechanisms by which it exerts these effects remain unknown.

Control of cell division and cell death during neurogenesis is critical for the generation of new neurons. Among other functions, the tumor suppressor protein p53 has dual roles in the regulation of cell cycle and apoptosis. P53 is a sequence-specific transcription factor that can regulate the expression of genes involved in a number of cellular processes including cell cycle checkpoint control, metabolism, autophagy and apoptotic cell death.^25^ Specifically, the cyclin-dependent kinase inhibitor p21 is a p53 target gene known to have a key role in p53-mediated cell cycle arrest.^26, 27^ P53 has also been shown to induce the expression of a number of genes involved in promoting apoptosis including Trp53INP1, Fas, Noxa and Puma (p53-upregulated modulator of apoptosis).^28^ Recent evidence suggests that P53 has a role in regulating neurogenesis in the developing and adult brain.^29^ Indeed, p53 expression is enriched in NPCs during development and in adult neurogenic regions such as the subventricular zone and subgranular zone.^30, 31^ Postnatal p53-deficient mice exhibit increased proliferation within the SVZ and increased neurogenesis.^30^ Furthermore, NPCs derived from *p53*-null mice exhibit reduced apoptosis and enhanced proliferation.^32^ However, the potential role of p53 in regulating NPC proliferation and survival during neuroinflammatory conditions has not been investigated.

In the present study, we demonstrate that IL-1*β* produced by lipopolysaccharide/interferon-*γ* (LPS/*γ*IFN)-activated microglia decreases NPC proliferation and survival. We show that IL-1*β* upregulates p53 and p53-mediated gene expression leading to cell cycle inhibition and Puma/Bax (Bcl-2-associated X protein)-mediated apoptosis in NPCs. Moreover, we demonstrate that p53-deficient NPCs are resistant to apoptosis and proliferation defects induced by microglia-derived IL-1*β*, suggesting that p53 is a key regulator of NPC proliferation and survival during neuroinflammatory conditions.

## Results

### Activated microglia-derived IL-1*β* induces cell cycle arrest and apoptosis in NPCs

**Figure 1:**
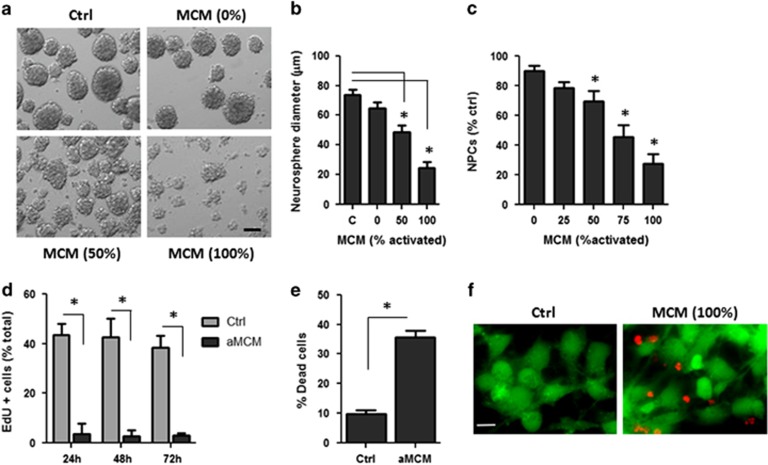
Conditioned media from LPS/*γ*IFN-activated microglia inhibits proliferation and induces cell death of NPCs. NPCs were cultured as neurospheres for 7 days in either unconditioned stem cell media (c, ctrl) or microglia-conditioned stem cell media from either unactivated microglia (0% MCM) or LPS/*γ*IFN-activated microglia (25–100% MCM). aMCM was left undiluted (100% MCM) or diluted to 75, 50 or 25% with unactivated MCM. (**a**) Representative images of NPCs grown as neurospheres for 7 days in naive stem cell media, unactivated MCM or increasing concentrations of aMCM. Scale bar: 50 *μ*m. (**b**) The mean diameter of neurospheres was measured after 7 days in culture in unconditioned or microglia-conditioned stem cell media (*n*=4, *P<0.05). (**c**) Neurospheres were dissociated after 7 days in culture and the number of NPCs were counted and reported as a percentage of NPCs obtained from neurospheres grown in control stem cell media (*n*=4, *P<0.05). (**d–f**) After 7 days in culture, neurospheres grown in complete stem cell media were dissociated and plated as a monolayer. NPCs were then incubated for indicated periods of time in complete stem cell media (ctrl) or conditioned stem cell media from a activated microglia (100% MCM). (**d**) To assess proliferation, NPCs were pulse labeled with EdU at the indicated times and counterstained with Hoechst 33342. NPCs were visualized by fluorescence microscopy and the fraction of Edu-positive cells was determined (*n*=4, *P<0.001). (**e**) The fraction of dead (ethidium-positive) NPCs was determined by Live/Dead assay following incubation with aMCM or unconditioned stem cell media (ctrl) for 72 h (*n*=3; *P<0.05). (**f**) Representative images of live (green)/dead (red) staining in NPCs. Scale bar: 10 *μ*m

NPCs isolated from the mouse telencephalon (E13.5) can be expanded *ex vivo* in stem cell media containing EGF/FGF to form neurospheres.^33^ To examine the effects of microglia-derived inflammatory factors on NPC proliferation, we cultured NPCs for 7 days in unconditioned neural stem cell media or conditioned stem cell media from either unactivated microglia or LPS/*γ*IFN-activated microglia. As shown in Figure 1, NPCs cultured in conditioned media from LPS/*γ*IFN-activated microglia produce significantly smaller neurospheres after 7 days in culture, as compared with those cultured in naive stem cell media or conditioned media from unactivated microglia (Figures 1a and b). Furthermore, the number of NPCs obtained following dissociation of the week-old neurospheres was also found to be significantly reduced following culture in activated microglia-conditioned media (aMCM) (Figure 1c). We next examined whether the decrease in proliferation was due to effects on cell cycle progression and/or cell death. To determine whether microglia-derived factors affected cell division, NPCs were grown as an adherent monolayer and pulse labeled with the nucleotide analog 5-ethynyl-2′-deoxyuridine (EdU). As shown in Figure 1d, the fraction of dividing (EdU-positive) cells was markedly reduced when NPCs were cultured in aMCM as compared with unactivated MCM. Furthermore, NPCs cultured in aMCM exhibited a significant increase in cell death as determined by Live/Dead assay (Figures 1e and f). These results suggest that proinflammatory microglia-derived factors inhibit NPC proliferation through the induction of both cell cycle arrest and cell death.

**Figure 2:**
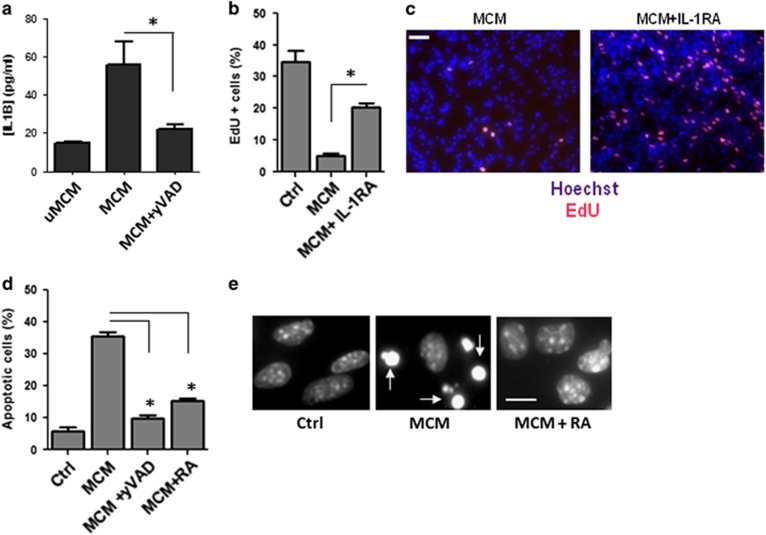
Microglia-derived IL-1*β* induces cell cycle arrest and apoptotic death of NPCs. (**a**) Conditioned stem cell media from unactivated MCM (uMCM), LPS/*γ*IFN-activated microglia (MCM) or microglia activated in the presence of the caspase-1 inhibitor y-VAD-CMK (20 *μ*M) was collected after 24 h and assayed for IL-1*β* levels by ELISA (*n*=3, *P<0.05). (**b**) NPCs were treated with unconditioned stem cell media (Ctrl) or conditioned media from LPS/*γ*IFN-activated microglia in the presence or absence of IL-1RA (50 ng/ml) for 72 h and then pulse labeled with EdU. NPCs were fixed and EdU was detected using AlexaFluor 594 azide and cells were counterstained with Hoechst 33342. The number of EdU-positive NPCs was counted and values are expressed as a percentage of the total number of cells (*n*=3; *P<0.01). (**c**) Representative images of EdU labeling of NPCs cultured in MCM in the presence or absence of IL-1RA (50 ng/ml). Scale bar: 50*μ*m. (**d**) NPCs were incubated for 72 h in unconditioned stem cell media (Ctrl) or LPS/*γ*IFN-aMCM in the presence or absence of y-VAD (20 *μ*M) or IL-1RA (50 ng/ml). NPCs were stained with Hoechst 33342 and the fraction of apoptotic nuclei was determined by examining the nuclei morphology (*n*=4; *P<0.01). (**e**) Representative images of Hoechst staining in NPCs treated with aMCM for 72 h in the presence or absence of IL-1RA (RA). Arrows highlight NPCs exhibiting apoptotic nuclear morphology. Scale bar: 10 *μ*m

Upon activation by proinflammatory stimuli, the microglia produce a number of soluble factors that can influence cells in the microenvironment, including TNF*α*, IL-6 and IL-1*β*.^13, 17, 18^ A number of studies have demonstrated that IL-1*β* in particular can inhibit neurogenesis *in vitro* and *in vivo*.^20, 21, 22, 23^ Therefore, we examined whether IL-1*β* contributed to the antiproliferative actions of microglia on NPCs using two different approaches. In the first approach, we added the caspase-1/ICE inhibitor, y-VAD-CMK (N-Ac-Tyr-Val-Ala-Asp-chloromethyl ketone), to microglia during LPS/*γ*IFN stimulation to prevent the processing and production of mature IL-1*β* (Figure 2a). In a second approach, we specifically blocked IL-1 R signaling in NPCs using the natural IL-1 receptor antagonist (IL-1RA) that competitively inhibits binding of IL-1*β* to the IL-1R1 receptor.^19, 34^ As shown in Figures 2b and c, the inhibitory effect of aMCM on NPC proliferation, as assessed by EdU labeling, was significantly reduced by treatment of NPCs with IL-1RA. Furthermore, we found that inhibition of IL-1*β* by either y-VAD or IL-1RA significantly reduced aMCM-induced NPC apoptosis (Figures 2d and e). These results indicate that IL-1*β* released by activated microglia induces both cell cycle arrest and apoptosis in NPCs.

### Microglia-derived IL-1*β* induces p53 activation in NPCs

**Figure 3:**
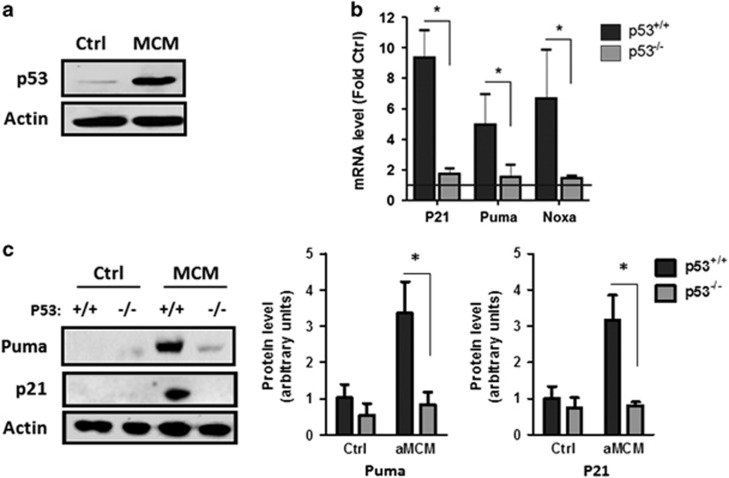
aMCM induces the expression of p53 and p53 target genes in NPCs. (**a**) NPCs were cultured in unconditioned stem cell media (Ctrl) or aMCM for 24 h and p53 protein levels were determined by western blot. A representative blot from three independent experiments is shown. (**b**) RNA was harvested from *p53*^+/+^ and *p53*^−/−^ NPCs incubated with MCM for 24 h and p21, Puma and Noxa mRNA levels were determined by quantitative real-time-polymerase chain reaction (qRT-PCR). mRNA levels are reported as fold increase over NPCs cultured in unconditioned stem cell media (*n*=4; *P<0.05). (**c**) Protein was extracted from *p53*^+/+^ and *p53*^−/−^ NPCs cultured for 48 h in either unconditioned stem cell media (Ctrl) or conditioned media from LPS/*γ*IFN-activated microglia (MCM) and Puma and p21 protein levels were determined by western blot. A representative immunoblot and densitometric analysis of immunoblots from three independent experiments is shown (*n*=3; *P<0.05)

The tumor suppressor p53 is a transcription factor that has been implicated in the regulation of genes involved in the control of cell cycle and apoptosis. P53 is expressed in NPCs and has been suggested to have a role in the regulation of neurogenesis in the adult brain.^29, 30, 32^ As our initial findings indicated that aMCM induces both cell cycle inhibition and cell death in NPCs we examined whether p53 was activated in NPCs under neuroinflammatory conditions. Protein levels of p53 were consistently increased in NPCs in response to treatment with aMCM (Figure 3a). We also observed marked increases in the expression of several p53 target genes known to be involved in the regulation of cell cycle arrest and apoptosis. *P21* is a cyclin-dependent kinase inhibitor and a known target gene of p53 that functions as a negative regulator of cell cycle progression at the G1–S phase.^26, 27^ Puma and Noxa are proapoptotic members of the Bcl-2 (B-cell lymphoma-2) gene family and are known to be transcriptionally regulated by p53.^35, 36, 37^ Interestingly, a robust increase in the expression of p21, Puma and Noxa mRNA was observed in NPCs treated with aMCM (Figure 3b). Consistent with their induction being mediated by p53, we found that the expression of p21, Puma and Noxa was not induced in p53-deficient NPCs (Figure 3b). Similarly, we found that p21 and Puma protein levels were upregulated in a p53-dependent manner in NPCs treated with aMCM (Figure 3c).

**Figure 4:**
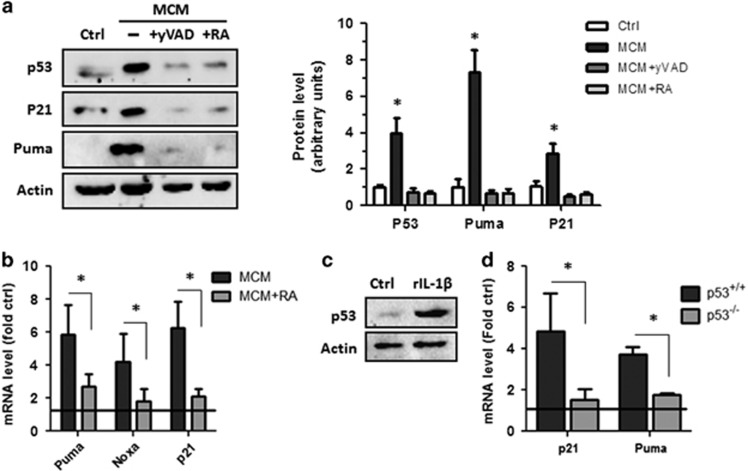
Microglia-derived IL-1*β* and rIL-1*β* induce p53 and p53 target gene expression in NPCs. NPCs were cultured in unconditioned stem cell media (Ctrl), conditioned media from microglia activated with LPS/*γ*IFN in the presence or absence of yVAD-CMK (20 *μ*M) or LPS/*γ*IFN-aMCM supplemented with IL-1RA (50 ng/ml). (**a**) NPCs were harvested after 48 h and protein extracts were subjected to sodium dodecyl sulfate polyacrylamide gel electrophoresis (SDS-PAGE) and immunoblotted for p53, Puma, p21 and actin as a loading control. Representative immunoblots and densitometric analysis of three independent experiments is shown (*n*=3; *P<0.05). (**b**) RNA was harvested after 24 h and mRNA levels of Puma, Noxa and p21 were determined by quantitative real-time-polymerase chain reaction (qRT-PCR). mRNA levels are reported as fold increase over NPCs incubated in unconditioned media (*n*=4, *P<0.05). (**c**) NPCs treated with recombinant IL-1*β* (50 ng/ml) were harvested after 48 h and subjected to SDS-PAGE and immunoblotted for p53 and actin as a loading control. A representative blot from three independent experiments is shown. (**d**) RNA was harvested from *p53*^+/+^ and *p53*^−/−^ NPCs treated with rIL-1*β* for 24 h and mRNA levels of Puma and p21 were examined by qRT-PCR. mRNA levels are reported as fold increase over control NPCs treated with vehicle (*n*=3; *P<0.01)

We next sought to determine whether microglia-induced p53 activation in NPCs was mediated by IL-1*β*. As shown in Figure 4a, inhibition of IL-1*β* signaling by either the caspase-1 inhibitor y-VAD-CMK or the IL-1RA blocked aMCM-induced p53 expression in NPCs. Inhibition of IL-1*β* production/signaling also attenuated the induction of the p53 target genes *p21* and *Puma* at both the mRNA and protein levels (Figures 4a and b). To further investigate the relationship between IL-1*β* and p53, we treated NPCs with recombinant IL-1*β* (rIL-1*β*) to determine whether this was sufficient to induce p53 activation. As shown in Figure 4c, rIL-1*β* treatment markedly increased p53 protein levels in NPCs. Furthermore, we found that rIL-1*β* induced p21 and Puma expression in *p53*^+/+^ but not *p53*^−/−^ NPCs (Figure 4d).

**Figure 5:**
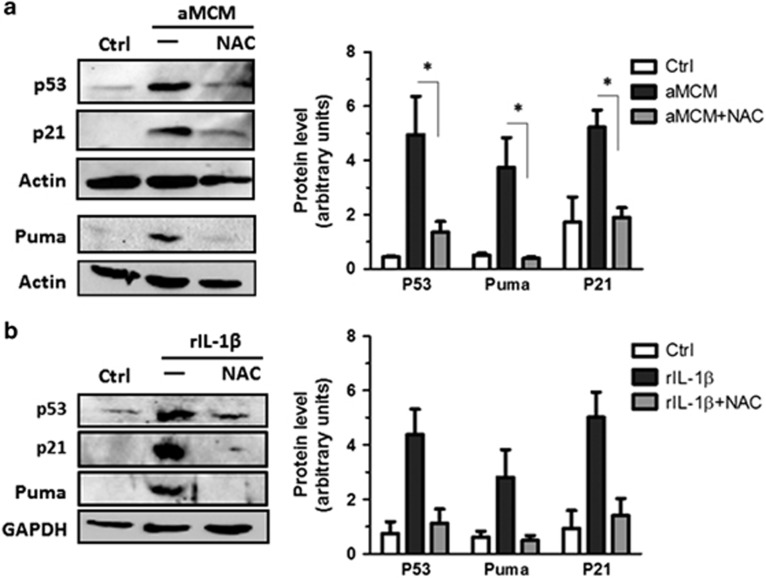
IL-1*β* activates p53 through an oxidative stress-dependent mechanism. (**a**) NPCs were cultured in unconditioned stem cell media (Ctrl) or LPS/*γ*IFN-aMCM in the presence or absence of *N*-acetylcysteine (5 mM). Protein was extracted at 48 h and p53, Puma, p21 and actin expression was assayed by western blot. Representative immunoblots and densitometric analysis from three independent experiments is shown (*n*=3; *P<0.05). (**b**) NPCs were treated with rIL-1*β* (50 ng/ml) or left untreated (Ctrl) and protein was extracted after 48 h and assayed for p53, p21 and glyceraldehyde 3-phosphate dehydrogenase (GAPDH) expression by western blot. Representative immunoblots and densitometric analysis from three independent experiments is shown (*n*=3, *P<0.05)

It has previously been reported that IL-1*β* can stimulate the production of reactive oxygen species (ROS) in retinal epithelial cells and pancreatic *β*-cells.^38, 39^ P53 is known to be activated in response to oxidative damage;^40^ therefore, we examined whether Il-1*β* triggers p53 activation in NPCs via an oxidative stress-dependent mechanism. Consistent with this, we found that the induction of p53 and its target genes *p21* and *Puma* by activated MCM and rIL-1*β* was markedly reduced in the presence of the ROS scavenger *N*-acetyl-cysteine (Figures 5a and b). Taken together, these results suggest that microglia-derived IL-1*β* induces p53 activation in NPCs via an oxidative stress-dependent mechanism.

### Microglia/IL-1*β*-induced cell cycle arrest and apoptosis in NPCs is mediated by p53

**Figure 6:**
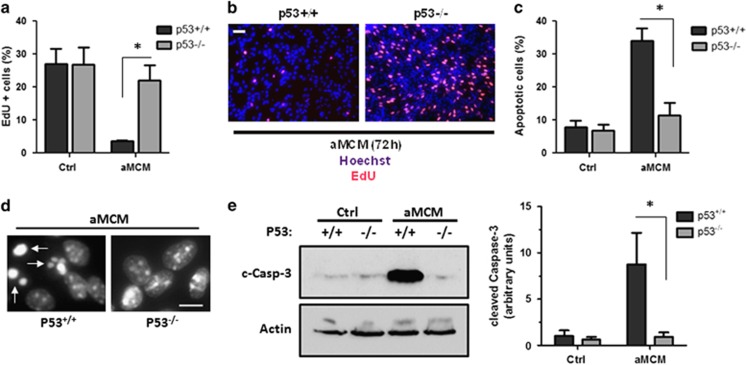
Activated microglia-induced cell cycle arrest and apoptosis in NPCs is mediated by p53. (**a**) *P53*^+/+^ and *p53*^−/−^ NPCs were cultured in unconditioned media (Ctrl) or aMCM for 72 h and then pulse labeled with EdU for 1 h. EdU was detected using AlexaFluor 594 azide and cells were counterstained with Hoechst 3342 nuclear dye. The number of EdU-positive cells were counted and expressed as a percentage of total cells (*n*=5, *P<0.01). (**b**) Representative images of EdU labeling of *p53*^+/+^ and *p53*^−/−^ NPCs cultured in aMCM for 72 h. Scale bar: 50 *μ*m. (**c**) *P53*^+/+^ and *p53*^−/−^ NPCs were incubated for 72 h in unconditioned stem cell media (Ctrl) or LPS/*γ*IFN-aMCM. NPCs were stained with Hoechst 33342 and the fraction of apoptotic nuclei was determined by examining nuclei morphology (*n*=4; *P<0.001). (**d**) Representative images of Hoechst staining in *p53*^+/+^ and *p53*^−/−^ NPCs incubated for 72 h in aMCM. Arrows highlight NPCs exhibiting apoptotic nuclear morphology. Scale bar: 10 *μ*m. (**e**) Protein was extracted from *p53*^+/+^ and *p53*^−/−^ NPCs 48 h after treatment with aMCM and active (cleaved) caspase-3 protein levels were determined by western blot. Representative immunoblot and densitometric analysis of three independent experiments is shown (*n*=3; *P<0.05)

We next examined whether p53 is required for the microglia-induced effects on NPC proliferation and cell death. Consistent with this, we found that aMCM induced a significant reduction in the fraction of EdU^+^ cells in *p53*^+/+^ NPC cultures but not in *p53*^−/−^ NPC cultures (Figures 6a and b). Moreover, we found that caspase-3 activation and apoptotic cell death induced by aMCM was markedly reduced in p53-deficient NPCs (Figures 6c–e).

**Figure 7:**
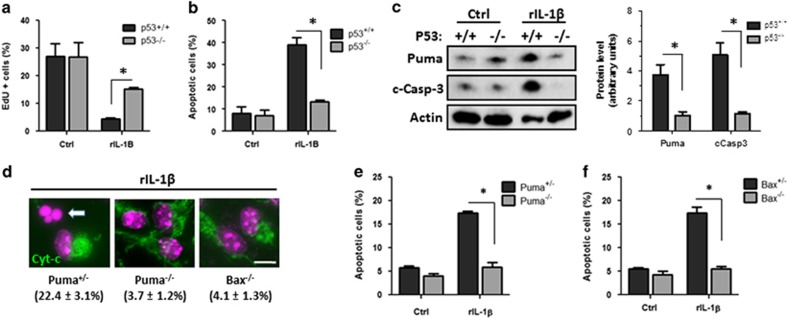
IL-1*β* is sufficient to inhibit proliferation and induce NPC apoptosis via p53-mediated induction of Puma. (**a**) *P53*^+/+^ and *p53*^−/−^ NPCs were treated with rIL-1*β* (50 ng/ml) and after 72 h NPCs were pulse labeled with EdU to detect dividing cells. The number of EdU-positive NPCs was counted and is reported as the percentage of the total number of cells (*n*=5; *P<0.05). (**b**) *P53*^+/+^ and *p53*^−/−^ NPCs were treated with rIL-1*β* (50 ng/ml) for 72 h and stained with Hoechst 33342. The fraction of apoptotic cells was determined by examining nuclear morphology (*n*=4, *P<0.01). (**c**) Protein was harvested from *p53*^+/+^ and *p53*^−/−^ NPCs treated with rIL-1*β* (50 ng/ml) for 48 h. Protein levels of Puma, active (cleaved) caspase-3 and actin as a loading control were examined by western blot. Representative immunoblots and densitometric analysis of four independent experiments is shown (*n*=4; *P<0.05). (**d**) Representative images of cytochrome *c* immunostaining (green) and Hoechst counterstaining (pseudocolored purple) in *Puma*^+/−^, *Puma*^−/−^ and *Bax*^−/−^ NPCs treated with vehicle or rIL-1*β* for 72 h. Arrow highlights an NPC exhibiting apoptotic nuclear morphology and lacking mitochondrial cytochrome *c* indicative of mitochondrial permeabilization. The percentage of NPCs exhibiting loss of mitochondrial cytochrome *c* for each genotype is indicated and represents the mean and S.D. (*n*=3, *P<0.01). Scale bar: 10 *μ*m. (**e**) *Puma*^+/−^ and *Puma*^−/−^ NPCs were treated with rIL-1*β* (50 ng/ml) for 72 h and the fraction of apoptotic cells was determined by Hoechst 33342 staining (*n*=4, *P<0.01). (**f**) *Bax*^+/−^ and *Bax*^−/−^ NPCs were treated with rIL-1*β* and the fraction of apoptotic cells was determined after 72 h by Hoechst 33342 staining (*n*=4, *P<0.01)

As we had found that rIL-1*β* was sufficient to activate p53, we examined whether p53 was required for rIL-1*β*-induced cell cycle arrest and apoptosis. Indeed, we found that rIL-1*β* treatment markedly reduced the fraction of EdU-labeled cells in wild-type NPC cultures and that *p53*^−/−^ NPCs were largely resistant to the antiproliferative effects of rIL-1*β* (Figure 7a). Furthermore, we found that rIL-1*β* induced caspase-3 activation and apoptosis in NPCs and that these effects were essentially abolished in *p53*-null NPCs (Figures 7b and c). Puma is a proapoptotic member of the Bcl-2 protein family and has been shown to function by promoting Bax-mediated mitochondrial permeabilization.^41^ We have found that Puma expression is induced by rIL-1*β* in a p53-dependent manner (Figure 7c), and consistent with the role of p53 in IL-1*β-*mediated cell death, we found that both *Puma*^−/−^ and *Bax*^−/−^ NPCs are resistant to rIL-1*β-*induced mitochondrial permeabilization as demonstrated by their maintenance of mitochondrial cytochrome *c* staining (Figure 7d). Furthermore, we found that rIL-1*β*-induced apoptosis was attenuated in both *Puma*^−/−^ and *Bax*^−/−^ NPCs (Figures 7e and f). Taken together, these results suggest that p53 has an essential role in regulating microglia-derived IL-1*β-*induced cell cycle inhibition and apoptosis in NPCs, and that IL-1*β* induces NPC apoptosis via the p53-mediated activation of a Puma/Bax-mediated mitochondrial pathway.

## Discussion

Neurogenesis occurs throughout life in two areas of the adult brain: the subventricular zone of the lateral ventricles and the dentate gyrus of the hippocampus.^42^ Neurogenesis has an important role in learning and memory^1, 2^ and deficits in adult neurogenesis have been implicated in the cognitive impairments observed in rodent models of Alzheimer's disease.^43, 44^ The persistence of neurogenesis in the adult brain also suggests the potential for regeneration and repair of the affected nervous system. Indeed, increased neurogenesis is observed following ischemic injury and status epilepticus, as well as in models of neurodegenerative disease.^6, 8, 45, 46^ However, neuroinflammatory processes associated with these neurological conditions have been shown to inhibit neurogenesis, thereby limiting the capacity for regeneration.^10, 11, 12, 13, 47^ Microglia are the primary regulators of neuroinflammatory responses and previous studies have demonstrated that activated microglia release proinflammatory cytokines such as TNF*α*, IL-1*β* and IL-6 and decrease the proliferation and survival of NPCs.^13, 17, 18^ However, the role and mechanism of action of specific microglia-derived proinflammatory cytokines on NPCs has not been clearly defined. Importantly, in the present study we demonstrate that LPS/*γ*IFN-activated microglia release the proinflammatory cytokine IL-1*β*, which has a pivotal role in inhibiting the proliferation and survival of NPCs. Specifically, we found that blocking IL-1*β* production in microglia using a caspase-1 inhibitor or blocking IL-1*β* signaling in NPCs with the receptor antagonist IL-1RA abrogated the effects of microglial on NPCs, resulting in restoration of proliferation and protection from apoptosis. Consistent with this, a number of studies have demonstrated that exogenous IL-1*β* can inhibit neurogenesis *in vitro* and *in vivo.* Both embryonic and adult NPCs express IL-1R1 and it has been demonstrated that recombinant IL-1*β* can decrease the proliferation of NPCs in culture,^20, 21^ and that hippocampal neurogenesis is impaired in mice chronically exposed to IL-1*β in vivo*.^22, 23^ Elevated levels of IL-1*β* and impaired hippocampal neurogenesis has also been observed in rodent models of chronic stress and it has been shown that the antineurogenic effects and behavioral symptoms induced by stress can be alleviated by IL-1RA or IL-1 receptor knockout.^22, 23^ In yet another study, it was shown that transplantation of IL-1RA-overexpressing NPCs into a mouse model of Alzheimer's disease rescued hippocampal neurogenesis and spatial memory disturbances.^48^ Taken together, these studies implicate IL-1*β* as an antineurogenic factor and suggest that targeting IL-1*β* may promote neurogenesis and enhance regenerative capacity in diverse neurological conditions.

NPCs are tightly regulated in terms of proliferation, self-renewal and survival processes. Recent studies demonstrate that p53 family members co-operate to regulate adult NPC pools.^29^ The p53 family of transcription factors consists of p53, p63 and p73. There are two major isoforms of p63 and p73: full-length transactivation-competent (TA) and N-terminally truncated (ΔN) isoforms that lack transcriptional activity and suppress the function of p53.^49, 50, 51^ The most predominantly expressed p53 family members in NPCs are p53, ΔNp63 and TAp73.^52, 53^ Studies have suggested that ΔNp63 promotes survival of NPCs by opposing the activation of proapoptotic p53 target genes, whereas TAp73 functions to promote self-renewal of NPCs.^52, 53, 54, 55^ Importantly, we have identified p53 as a negative regulator of NPC proliferation and survival in response to neuroinflammatory factors. Specifically, we demonstrate that microglial-derived IL-1*β* as well as recombinant IL-1*β* induce p53 expression in NPCs and trigger a p53-dependent increase in the expression of the cell cycle regulator p21. Studies have shown that p21 deficiency results in increased NPC proliferation in the lateral ventricle wall of adult mice, and in the hippocampus and subventricular zone following ischemic injury.^56, 57^ Interestingly, a recent study found that haploinsufficiency of the p53 family member p73, or combined haploinsufficiency of p63 and p73, lead to increased levels of p21 and cellular senescence under basal conditions and more markedly following genotoxic stress.^55^ P73-deficient mice also possess fewer NPCs and exhibit dysregulated Sox2 and Notch signaling, as well as increased cellular senescence, suggesting p73 has a major role in NPC self-renewal and proliferation.^52, 53^ These results suggest that p53 family members may work cooperatively or independently to regulate NPC pools. Thus, it would be interesting to determine whether the p53 family members p63 and p73 also have a role in the regulation of NPC proliferation and survival during neuroinflammatory conditions.

P53 activation can trigger the activation of apoptosis in a cell-type- and stimulus-specific manner via transcriptional regulation of proapoptotic Bcl-2 family members, APAF-1, DR5 and Fas, as well as by transcription-independent mechanisms.^28, 58^ Here we show that exogenous or microglial-derived IL-1*β* leads to increased expression of the proapoptotic BH3-only Bcl-2 family member Puma in a p53-dependent manner. Similar to our findings, IL-1*β* was also shown to induce the expression of Puma in pancreatic *β*-cells, although this appeared to occur through an NF-κB-dependent but p53-independent mechanism.^59^ Importantly, we demonstrate that deletion of Puma in NPCs confers significant protection from IL-1*β*-induced apoptosis. As further evidence of intrinsic (mitochondrial) apoptosis activation, Bax-deficient NPCs were also found to be resistant to IL-1*β*-induced death. Taken together, these results indicate that IL-1*β* induces apoptosis through the mitochondrial pathway of apoptosis via p53-mediated upregulation of Puma. In the present study, we have delineated this signaling pathway using cultured embryonic NPCs; however, it is possible that that specific adult NPC populations may respond differently in an *in vivo* environment. Therefore, in future studies, it will be interesting to determine whether targeting the IL-1*β*-p53 pathway affects NPC proliferation and survival in neurogenic regions of the adult brain during neuroinflammatory conditions *in vivo*.

In the present study, we identify a novel link between IL-1*β* and p53 activation in the regulation of NPC proliferation and survival. P53 is known to be activated by oxidative stress^40^ and IL-1*β* has been reported to induce ROS production in retinal epithelial cells through NADPH-oxidase activation and in pancreatic *β*-cells via induction of iNOS.^38, 39^ Consistent with this, we found that p53 activation by exogenous and microglia-derived IL-1*β* was abrogated in the presence of the ROS scavenger *N*-acetylcysteine. Activation of IL-1R1 by IL-1*β* is also known to activate several signaling pathways that have been implicated in the antineurogenic effects of IL-1*β* including JNK, GSK3*β* and NF-*κ*B.^21, 60, 61^ It is unclear whether these pathways affect p53 activation or co-operate with p53 to affect NPC proliferation and cell death. However, previous studies have demonstrated that both JNK and GSK3*β* can regulate p53 activity.^62, 63^ Furthermore, NF-*κ*B has been shown to co-operate with p53 to regulate the induction of proapoptotic factors.^64, 65^

In summary, we have identified a novel signaling pathway that regulates neuroinflammation-induced decreases in NPC proliferation and survival. Importantly, we identify the transcription factor p53 as a critical mediator of NPC regulation by IL-1*β* leading to cell cycle arrest and apoptosis. This could potentially provide targets to promote neurogenesis and repair in the CNS following injury or neurodegenerative pathology.

## Materials and Methods

### Animals

Mice carrying a targeted null mutation for Bax were obtained from Jackson Laboratories (Bar Harbor, ME, USA) and were genotyped as described previously.^66^ Mice carrying a targeted null mutation for Puma were generated and maintained on a C57/BL6 background in the laboratory of Dr. Andreas Strasser (WEHI, Melbourne, VIC, Australia). Genotyping of these mice was performed as described previously. Timed pregnant wild-type CD1 mice were purchased from Charles River Laboratories (Sherbrooke, QC, Canada).

### NPC culture

NPCs were dissociated from the telencephalon of E13.5 mice and grown as neurospheres for 7 days in neural stem cell media consisting of DMEM-F12 containing d-glucose (6 mg/ml), l-glutamine (2 mM), penicillin/streptomycin, insulin (20 mg/ml), apotransferrin (100 mg/ml), progesterone (0.02 nM), putrescine (20 nM), sodium selenite (30 nM), heparin (0.3 nM) and basic fibroblast growth factor-2 (bFGF) (10 ng/ml) as described previously.^33^ Neurospheres were then dissociated by incubation in 0.05% trypsin-EDTA and trituration with a glass pipette. Trypsin inhibitor was added and the single-cell suspension was centrifuged at 300x*g* for 5 min. Single-cell NPCs were plated on dishes coated with poly-l-ornithine and laminin (Sigma, Oakville, ON, Canada) at a density of 60 000 cells per cm^2^.

### Microglia cell culture and preparation of MCM

The mouse microglial cell line EOC-20 was obtained from the American Type Culture Collection (ATCC CRL-2469; ATCC, Manassas, VA, USA). Cells were maintained at 37 °C and 5% CO_2_ in DMEM supplemented with 10% fetal bovine serum, 0.5% penicillin/streptomycin, 4 mM l-glutamine and 20% conditioned medium from bone-marrow-derived Ladmac cells (ATCC CRL-2420) as a source of colony-stimulating factor-1. For preparation of MCM, EOC-20 cells were grown to 60% confluence at which point their media were removed and replaced with neural stem cell media (lacking bFGF and heparin). To activate microglia, stem cell media were supplemented with 10 ng/ml LPS (Sigma, Mississauga, ON, Canada) and 10 ng/ml recombinant mouse *γ*IFN (R&D Systems, Minneapolis, MN, USA) for 24 h. MCM was collected, centrifuged and filtered through a 0.2 *μ*m filter to remove cells and debris. MCM was then supplemented with 10 ng/ml bFGF and 0.3 nM heparin and immediately used for NPC cultures. In the indicated experiments, LPS/*γ*IFN was added to non-aMCM or unconditioned stem cell media immediately before adding to NPC culture.

### Neurosphere size quantification

NPCs were cultured for 7 days in suspension to form neurospheres in unconditioned stem cell media or conditioned stem cell media from either unactivated or LPS/*γ*IFN-activated microglia. Phase-contrast micrographs were taken at × 100 total magnification using a microscope equipped with a digital camera. Five representative images were taken for each treatment and analysis was carried out using Northern Eclipse 7.0 software (Empix Imaging Inc., Mississauga, ON, Canada) by manually measuring the diameters of neurospheres using the straight line measurement tool, which provides an arbitrary pixel length. Pixel length was then converted to *μ*m using the Calibrate for Distance tool. Two hundred spheres were measured per treatment and results are representative of three separate experiments.

### NPC treatments and IL-1*β* neutralization experiments

NPCs were treated with MCM or recombinant mouse IL-1*β* (rIL-1*β*; R&D Systems) 2 days after plating as a monolayer. Unless otherwise indicated, aMCM refers to 100% aMCM. In the indicated experiments, 50 ng/ml recombinant mouse IL-1RA (R&D Systems) was added to NPC cultures simultaneously with the switch to MCM or rIL-1*β* treatment. For inhibition of IL-1*β* cleavage in microglia, 20 *μ*M of the caspase-1 inhibitor y-VAD-CMK (Sigma) was added to microglia at the time of activation, and conditioned media was collected after 24 h.

### IL-1*β* ELISA

Conditioned stem cell media from microglia either left unstimulated or stimulated with 10 ng/ml LPS (Sigma)/10 ng/ml *γ*IFN was collected at 24 h. IL-1*β* levels were detected using the mouse IL-1*β* ELISA-Max (BioLegend, San Diego, CA, USA) as per the manufacturer's instructions. Briefly, microglia-conditioned media samples were added to microplates precoated with mouse polyclonal IL-1*β* antibody. Following incubation and washes to remove unbound IL-1*β*, an enzyme-linked mouse polyclonal antibody was added. The addition of the substrate yields a colorimetric product and the absorbance (450 nm) was measured using a microplate reader. Samples were assayed in duplicate and IL-1*β* concentrations were determined from a standard curve using the SoftmaxPro software (Molecular Devices, Sunnyvale, CA, USA).

### EdU-labeling experiments

Proliferation studies were performed by EdU labeling using the Click-iT EdU AlexaFluor 594 Imaging Kit (Invitrogen, Carlsbad, CA, USA) as per the manufacturer's protocol. Briefly, monolayer NPCs were labeled with EdU for 1 h before fixation with 4% paraformaldehyde. Cells were then washed two times with PBS containing 3% BSA, and permeabilized with 0.5% Triton X-100 in PBS. Cells were then incubated with ClickIT reaction cocktail containing AlexaFluor 594 azide for the detection of EdU labeling. Cells were counterstained with Hoechst 33258 (1 *μ*g/ml), and images were captured using fluorescence microscopy. A minimum of 400 cells per well were counted and the number of EdU-labeled cells was calculated as a fraction of total cells.

### Cell death assays

Apoptosis of NPCs was assessed by examining nuclear morphology in Hoechst 33342-stained cells as described previously.^67^ Briefly, NPCs were stained with 1 *μ*g/ml Hoechst 33342 (Sigma) and the fraction of cells exhibiting an apoptotic nuclear morphology characterized by chromatin condensation and/or apoptotic bodies was quantified. In certain experiments, NPC death was determined by Live/Dead assay according to the manufacturer's instructions (Invitrogen). Briefly, NPCs were stained with Calcein-AM (2 *μ*M) and ethidium homodimer (4 *μ*M) for 20 min and the fraction of live (Calcein-AM-positive) and dead (ethidium-positive) cells was scored. NPCs were visualized by fluorescence microscopy (Zeiss, Toronto, ON, Canada) and images were captured with a Zeiss Axio-Cam camera (Zeiss). Images were captured and scored by an observer blinded to the treatment. A minimum of 500 cells from five randomly selected fields were analyzed for each treatment and the fraction of apoptotic or dead cells was determined.

### Quantitative real-time RT-PCR

RNA was isolated using Trizol reagent as per the manufacturer's instructions (Invitrogen) and 10ng of RNA was used in one-step Sybr green RT-PCR (QuantiFast; Qiagen, Mississauga, ON, Canada). RT-PCR was carried out on a Chromo4 system (MJ Research Bio-Rad, Mississauga, ON, Canada) and changes in gene expression were determined by the Δ(ΔCt) method using S12 transcript for normalization. Data are reported as fold increase in mRNA levels in treated samples relative to untreated control cells. All PCRs exhibited high amplification efficiency (>90%) and the specificity of PCR products was confirmed by sequencing.

### Western blot and densitometric analysis

Whole-cell lysates were prepared by incubating NPCs in lysis buffer (150 mM NaCl, 1% NP-40, 0.5% sodium deoxycholate, 0.1% SDS, 50 mM Tris (pH 8), 1 mM EDTA, 1 mM DTT and protease and phosphatase inhibitor cocktail (Invitrogen)) for 20 min on ice. The soluble extract was recovered by centrifugation at 14,000x*g*. Protein concentration was determined by BCA assay (Pierce, Rockford, IL, USA) and 50 *μ*g of protein was separated on 12.5% SDS-PAGE gels and then transferred to nitrocellulose membranes. Membranes were blocked for 1 h in TBS-T (10 mM Tris, 150 mM NaCl, 0.05% Tween-20), followed by overnight incubation in primary antibodies to p53, cleaved (active) caspase-3, GAPDH (Cell Signaling Technology, Danvers, MA, USA), Puma (Sigma), p21 or actin (Santa Cruz Biotech, Santa Cruz, CA, USA) in TBS-T containing 5% skim milk. Membranes were washed with TBS-T and incubated for 1 h with the appropriate HRP-conjugated secondary antibodies and developed by enhanced chemiluminescence system according to the manufacturer's instructions (Bio-Rad, Mississauga, ON, Canada). Chemiluminescence signal was detected with a Bio-Rad ChemiDoc MP imaging system and densitometric measurements were determined using ImageLab software (Bio-Rad). Protein level was normalized to corresponding actin level and is reported as relative density units for protein of interest.

### Cytochrome *c* immunostaining

NPCs were fixed in 4% paraformaldehyde, washed in three changes of PBS and then incubated overnight with a monoclonal antibody directed against cytochrome *c* (BD PharMingen, San Diego, CA, USA). Cells were then washed and incubated for 1 h with AlexaFluor 488-conjugated goat anti-mouse IgG secondary antibody (Invitrogen) and counterstained with Hoechst 33258 (1 *μ*g/ml). To evaluate mitochondrial membrane permeabilization, cells were visualized by fluorescence microscopy and cells exhibiting punctate, cytoplasmic cytochrome *c* staining were considered to have maintained membrane integrity, whereas cells lacking cytoplasmic cytochrome *c* staining were considered to have undergone mitochondrial membrane permeabilization as we have described previously.^67^ Images were captured and scored by a blinded observer and a minimum of 400 cells were analyzed per well.

### Data analysis

Data are reported as mean and S.E.M. The ‘*n*' value represents the number of independent experiments and/or number of mice from which independent NPC cultures were prepared. Data were analyzed by one-way ANOVA followed by Tukey's *post hoc* test and differences were considered significant at *P*<0.05. All statistical analysis were conducted using GraphPad Prism software (GraphPad Software Inc., La Jolla, CA, USA).

## Abbreviations

Bax: Bcl-2-associated X protein
Bcl-2: B-cell lymphoma-2
bFGF: basic fibroblast growth factor-2
EdU: 5-ethynyl-2′-deoxyuridine
*γ*IFN: interferon-γ
IL-1*β*: interleukin-1*β*
IL-1R1: interleukin-1 receptor type 1
IL-1RA: interleukin-1 receptor antagonist
LPS: lipopolysaccharide
MCM: microglia-conditioned media
NPC: neural precursor cell
Puma: p53-upregulated modulator of apoptosis
rIL-1*β*: recombinant interleukin-1*β*
